# Establishment of an ES Cell-Derived Murine Megakaryocytic Cell Line, MKD1, with Features of Primary Megakaryocyte Progenitors

**DOI:** 10.1371/journal.pone.0032981

**Published:** 2012-03-02

**Authors:** Hedia Chagraoui, Catherine Porcher

**Affiliations:** MRC Molecular Haematology Unit, Weatherall Institute of Molecular Medicine, John Radcliffe Hospital, University of Oxford, Oxford, United Kingdom; Northwestern University, United States of America

## Abstract

Because of the scarcity of megakaryocytes in hematopoietic tissues, studying megakaryopoiesis heavily relies on the availability of appropriate cellular models. Here, we report the establishment of a new mouse embryonic stem (ES) cell-derived megakaryocytic cell line, MKD1. The cells are factor-dependent, their cell surface immunophenotype and gene expression profile closely resemble that of primary megakaryocyte progenitors (MkPs) and they further differentiate along the megakaryocyte lineage upon valproic acid treatment. At a functional level, we show that ablation of SCL expression, a transcription factor critical for MkP maturation, leads to gene expression alterations similar to that observed in primary, *Scl-excised* MkPs. Moreover, the cell line is amenable to biochemical and transcriptional analyses, as we report for *GpVI*, a direct target of SCL. Thus, the MKD1 cell line offers a pertinent experimental model to study the cellular and molecular mechanisms underlying MkP biology and more broadly megakaryopoiesis.

## Introduction

The study of megakaryopoiesis has been hindered by difficulties in obtaining large numbers of pure megakaryocytes. Although recent technical advances allow purification of megakaryocytes from primary hematopoietic progenitors [1]–[3], cell numbers remain low and the cultures are not synchronous nor homogeneous.

Permanent cell lines are an alternative source of megakaryocytes (MKs) and have proven very useful in studying megakaryocytic differentiation (for review, see Saito [4]). So far, MK cell lines have been mainly derived from human leukemic patients, with many inherent shortcomings including abnormal karyotypes. Moreover, many of these cell lines are immature and show multiphenotypic characteristics of erythroid, myeloid and MK lineages. Very few present with more restricted MK-specific phenotype and treatment with phorbol esters often enhances their MK program [4].

Here, we report the establishment of new MK cell lines from murine embryonic stem (mES) cells, upon expression of the homeobox gene *Hox-11*, a potent immortalizing agent of bone marrow [5] and ES cell-derived hematopoietic cells [6]. Several growth factor-dependent clones exhibiting megakaryocytic features and various degrees of megakaryocytic differentiation were obtained. Clone MKD1 was extensively studied at cellular, molecular and functional levels.

## Materials and Methods

### Mice

*Scl^fl/fl^* and *PF4-Cre* mice described in [7] were housed according to national and institutional guidelines for humane animal care.

### Immortalization of mouse *Scl^fl/fl^* ES cell-derived hematopoietic cells with *Hox11*

*Scl^fl/fl^* ES cells were obtained from neo-excised *Scl^fl/wt^* cells (derived from mouse E14Tg2a ES cells) [7] after a second round of homologous recombination. Transduction of *Scl^fl/fl^* ES cells with *Hox11* (*Hox11* vectors were gifts from G. Keller), differentiation into embryoid bodies (EBs) and establishment of immortalized cultures have been described [6].

### Antibodies and reagents

All antibodies were purchased from BD pharmingen except for CD42b, (PE-conjugated GpIbα, a kind gift from B. Nieswandt (Germany). Valproic acid was from Sigma.

Cellular staining, ploidy, FACS analyses, real-time PCR, MkP purification, nucleofection and Cre-mediated excision in MKD1 were as described [7].

#### GpVI promoter

A 330 bp sequence encompassing the *Gp6* promoter (−330/+1) was PCR-amplified from mouse genomic DNA and cloned into pGL4b (Promega). Luciferase-based transactivation assays were performed in 3T3 and MKD1 cells as described [7], [8]. For Chip, primers and 5′FAM-3′TAMRA labelled probes were selected from unique sequences in the *GpVI* locus and appropriate external controls using Primer Express Software (sequences available upon request). Input and immunoprecipitated material were analysed in duplicates relative to a sequence in the *Gapdh* locus.

#### Differentiation

Cells were seeded at a density of 2–4×10^5^ cells/ml in presence of Epo, IL-3 and valproic acid for 3 to 7 days.

## Results and Discussion

In an attempt to study the functional role of SCL/Tal1, a master regulator of hematopoiesis (see [7] and references therein), in ES cell-derived megakaryopoiesis, we generated *Scl^flox/flox^* ES cells. Importantly, using *in vitro* differentiation assays, we did not observe morphological or biological differences between wild-type and *Scl^flox/flox^* ES cell-derived hematopoietic cells and, more specifically MKs (data not shown), thereby establishing the neutrality of the loxP sites introduced into the *Scl* locus.

**Figure 1:**
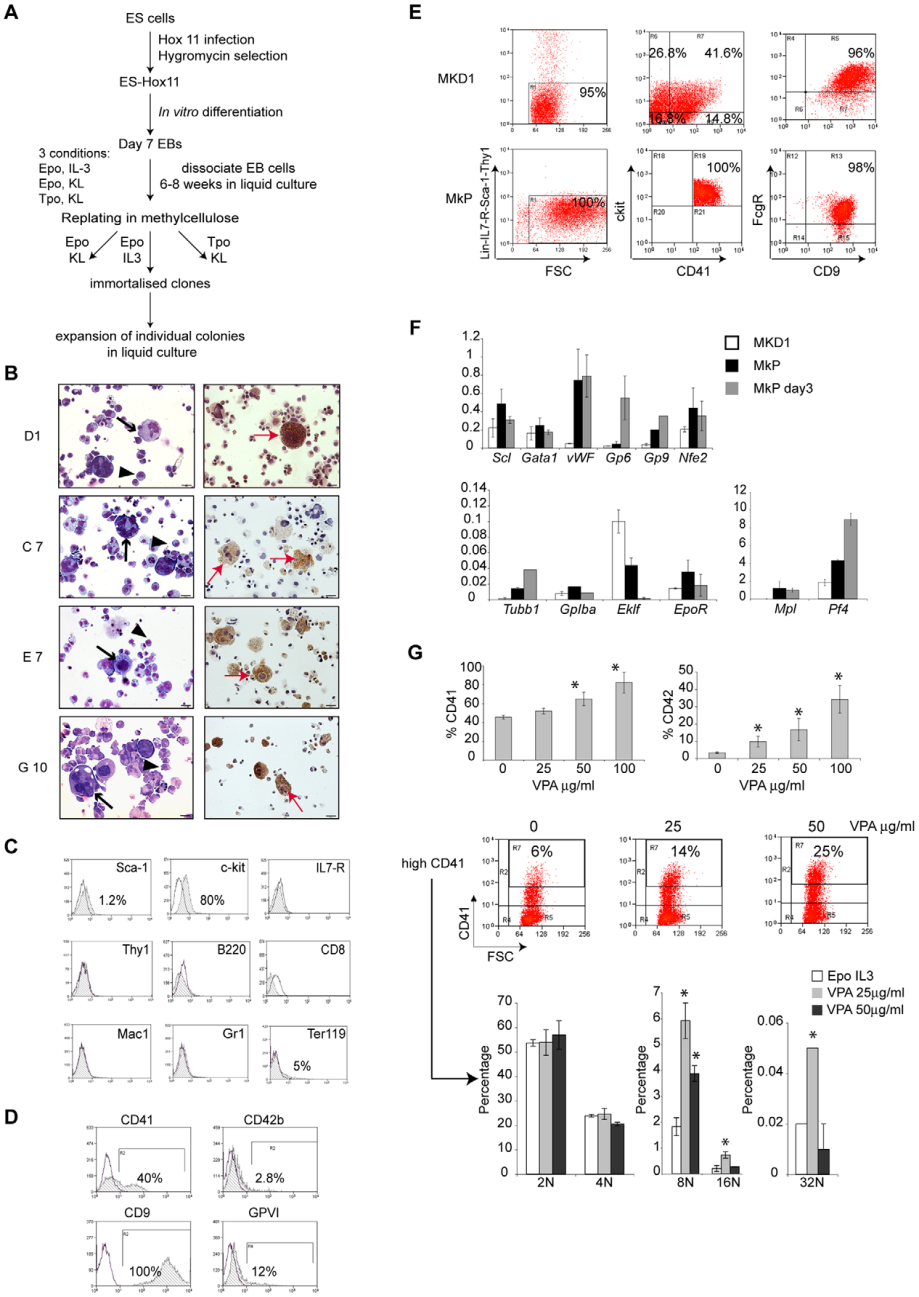
MKD1 cell line exhibits characteristics of primary MkPs. (**A**) Outline of the strategy used to isolate immortalized MK clones. (**B**) (Left) MGG staining reveals small cells with a high nuclear/cytoplasmic ratio (arrow heads) and large cells with multilobulated nuclei (arrows). (Right) AchE staining (red arrow). The photographs were taken using an Olympus BX60 microscope with a Qimaging camera (Surrey, BC). Openlab version 3 software (Improvision, Coventry, UK) was used for image acquisition and images were exported into Adobe Photoshop version CS2 (Adobe Systems, San Jose, CA). (**C–D**) Analysis of MKD1 cells by flow cytometry. Cells were stained for lineage-specific markers (C) and MK-specific markers (D). The hatched histograms represent the staining with the indicated antibody and the open histograms correspond to the isotype control. One representative experiment out of 3 is shown. (**E**) Comparison of the FACS-profile of MKD1 cells (top) to that of primary MkPs (bottom), defined as Lin^−^ sca1^−^IL7-R^−^ Thy1^−^, ckit^+^CD41^+^, Fcγ^low^ CD9^+^. (**F**) Gene expression profile in MKD1 cells. Analysis by real-time RT-PCR of levels of expression of MK and erythroid-specific markers from mRNA isolated from MKD1 (white bars), primary MkPs (black bars) and Day3 MkPs cultivated with a cocktail of cytokines (grey bars). The *y*-axis represents enrichment in cDNA sequences normalised to *Gapdh* gene control sequences. The data show the mean ± SD of 3 independent experiments. (**G**) Valproic acid (VPA)-induced differentiation of MKD1 cells. Percentages of CD41^+^ (Top left) and CD42b^+^ (Top right) MKD1 cells after 3 days of VPA treatment (25, 50 and 100 µg/ml). The histograms represent the mean ± SD of 3 independent experiments. *, p<0.05. (Middle) Facs plots showing the percentage of high CD41 expressing cells before and after VPA treatment (25 and 50 µg/ml). (Bottom) Ploidy analysis of MKD1 cells after VPA treatment for 7 days. The cells were gated on CD41 high expressing cells. The histograms represent the percentage of the different class ploidy for each condition (white bars: Epo, IL3, grey bars: Epo, IL3, VPA 25 µg/ml, black bars: Epo, IL3, VPA 50 µg/ml). The data show the mean ± SD of 3 independent experiments. *, p<0.002.

Hematopoietic cell lines were then established from *Scl^flox/flox^* ES cells (Figure 1A). Briefly, Hox-11 transduced ES cells were *in vitro* differentiated into embryoid bodies (EBs). Day 7 EBs were dissociated and cells maintained in liquid cultures in three different cytokine conditions (Epo/IL3, Tpo/KL, and Epo/KL). After 6 and 8 weeks, hematopoietic cells were seeded onto methylcellulose. Immortalized colonies were isolated 8 to 10 days later and expanded in liquid culture in the appropriate cytokine condition. Morphological inspection and immuno-phenotyping identified megakaryocytic (MK) cell lines in the Epo/IL3 condition only (not shown). In agreement with this, most Hox11-immortalized hematopoietic clones are IL3-dependent for their growth and survival [5]. Several immortalised MK clones showing different degrees of differentiation were obtained, as judged by cellular staining (MGG and Acetylcholine Esterase, AchE, a MK-specific marker) (Figure 1B) and by the percentage of cells that (i) express CD41^+^ (GpIIb) and CD42b^+^ (GpIbα) (two cell surface markers expressed in differentiating MKs); (ii) are positive for AchE; (iii) exhibit ploidies greater than 8N (Table 1). Whilst clones C7, E7 and G10 showed relatively high levels of CD41 and CD42b expression (ranging from 67% to 96% and 12% to 30%, respectively) and AchE positivity (15% to 90%), clone D1 (MKD1) showed lower CD41, CD42b and AchE expression (40%, 2.8% and 2% respectively). Although clones E7 and G10 seemed more advanced in their differentiation than the MKD1 clone, their ploidy (4% of E7 and G10 cells had a ploidy greater than 8N, compared to 3% for the MKD1 clone) and their gene expression profile, when assayed for the MK-specific markers shown in Figure 1F (data not shown), were similar to that of MKD1 cells. We decided to focus on the MKD1 clone as a potential model of early megakaryopoiesis because, similarly to MkPs and immature MKs, MKD1 displayed low AchE activity and low ploidy [9]. Moreover, as described below, expression of CD41 and 42b increases as MKD1 cells differentiate along the megakaryocytic lineage, as also reported for MkPs [9].

**Table 1:** Generation of MK clones showing different degrees of differentiation.

Clones	CD41 (%)	CD42b (%)	AchE (%)	Ploidy>8N (%)
C7	67	12	15	2.2
E7	96	30	50	4.27
G10	96	24	90	4.04
D1	40	2.8	2	3.03

MKD1 cells exhibited a MK-specific immunophenotype (Figure 1 C–D). They were negative for the early stem cell marker Sca1 (1.2%) and for markers of lymphoid (B220, CD8, Thy1, IL7-R) and myeloid (Gr1, Mac1) lineages; although 5% of the cells expressed Ter119 (erythroid-specific), they did not stain for benzidine (not shown), suggesting limited erythroid potential. They essentially expressed ckit and MK-specific markers CD41/CD61 (40 to 50%), CD42b (2.8%), GPVI (12%) and CD9 (100%) (Figure 1D). In order to better assign them to a specific stage in megakaryopoiesis, we compared MKD1 cells to primary megakaryocyte progenitors (MkPs) as defined by Weissman et al [2] (Figure 1E). Similarly to MkPs, MKD1 cells were mostly ckit^+^, CD9^+^, FcγRII/III^low^, but only 50 to 60% CD41^+^ as opposed to 100% in MkPs.

Next, we compared the levels of expression of selected genes in MKD1 cells, to that observed in MkPs and more mature, MkP-derived MKs (MkPs day3) (Figure 1F). Expression of the genes coding for the transcription factors *Scl*, *Gata1*, *Nfe2* and the surface membrane protein *GpIbα* was similar across the cell types; *Eklf* was expressed at higher levels in MKD1 than in MkPs and not detected in mature MKs. Confirming the immature nature of MKD1 cells, levels of late markers such as β1-tubulin (*Tubb1*), Glycoprotein 9 (*Gp9*), von Willebrand factor (*vWF*) and Platelet factor 4 (*Pf4*) were lower in MKD1 than MkPs; Glycoprotein 6 (*Gp6*) was expressed at low levels, similar to that in MkPs. Interestingly, compared to MkPs, MKD1 cells express similar levels of *Epo* receptor (*EpoR*), but very low levels of the thrombopoietin (*Tpo*) receptor (*cMpl*), consistent with their EPO-dependence. EpoR and cMPL, as well as their respective ligands, share significant structural [10] and functional homology [11], [12]. Therefore, although TPO remains the primary growth factor of the MK lineage [13], supporting both proliferation and differentiation of MkPs, Epo/EpoR-mediated signalling pathways could substitute for those normally transmitted through Tpo/cMPL in MKD1 cells.

It is noteworthy to point out that MKD1 cell line was derived from day 7 EBs, a stage representative of the yolk sac phase of hematopoiesis [14]. Interestingly, Xu et al. [15] have reported the existence in the yolk sac of MKs with specific features when compared to adult bone marrow MKs, such as lower ploidy and different responsiveness to cytokines: Epo and SCF stimulate the formation of MK colonies derived from early yolk sac but not adult bone marrow cells [15]. We propose that an early (yolk sac type) origin of MKD1 cells could therefore explain why Epo sustains their growth. Albeit at low levels, the MKD1 clone expresses the erythroid marker Ter119 (Figure 1C). Therefore, to be able to distinguish between the primitive versus definitive nature of the MKD1 cells (corresponding to the first versus second wave of hematopoiesis in the yolk sac, see [16]), we assessed globin gene expression (data not shown). Interestingly, the MKD1 cells did not express embryonic *βH1* globin, but we did detect expression of the adult α and β globin genes. Altogether, these data suggest that MKD1 cells are likely to represent the second wave of yolk sac hematopoiesis giving rise to definitive progenitors [16].

Overall, the cell surface immunophenotype, ploidy and mRNA content show that MKD1 cells share similarities with MkPs. The immature phenotype of MKD1 could be explained by two factors. First, *Hox11* enforced expression is indeed associated with immature phenotypes [5]; second, IL3 is known to promote the earliest stage of megakaryopoiesis while inhibiting further maturation after endomitosis begins [17].

We then investigated whether MKD1 cells could further differentiate along the MK pathway. We did not see any difference in CD41/CD42 expression and ploidy levels at high concentrations of TPO (100 ng/ml) or phorbol myristic acetate (TPA) (not shown). In contrast, treatment of MKD1 cells with valproic acid (VPA), a potent inhibitor of histone deacetylases recently reported to promote megakaryopoiesis [18]–[20] increased CD41 and CD42 expression in a dose-dependent manner (Figure 1G, top). Moreover, the CD41^high^ cell population (increased by 2 to 4-fold upon VPA treatment) (Figure 1G, middle) showed a 3-fold increase in the 8N, 16N and 32N ploidy classes at 25 µg/ml of VPA (Figure 1G, graphs). Therefore, VPA induces MKD1 cells to differentiate further along the MK pathway.

**Figure 2:**
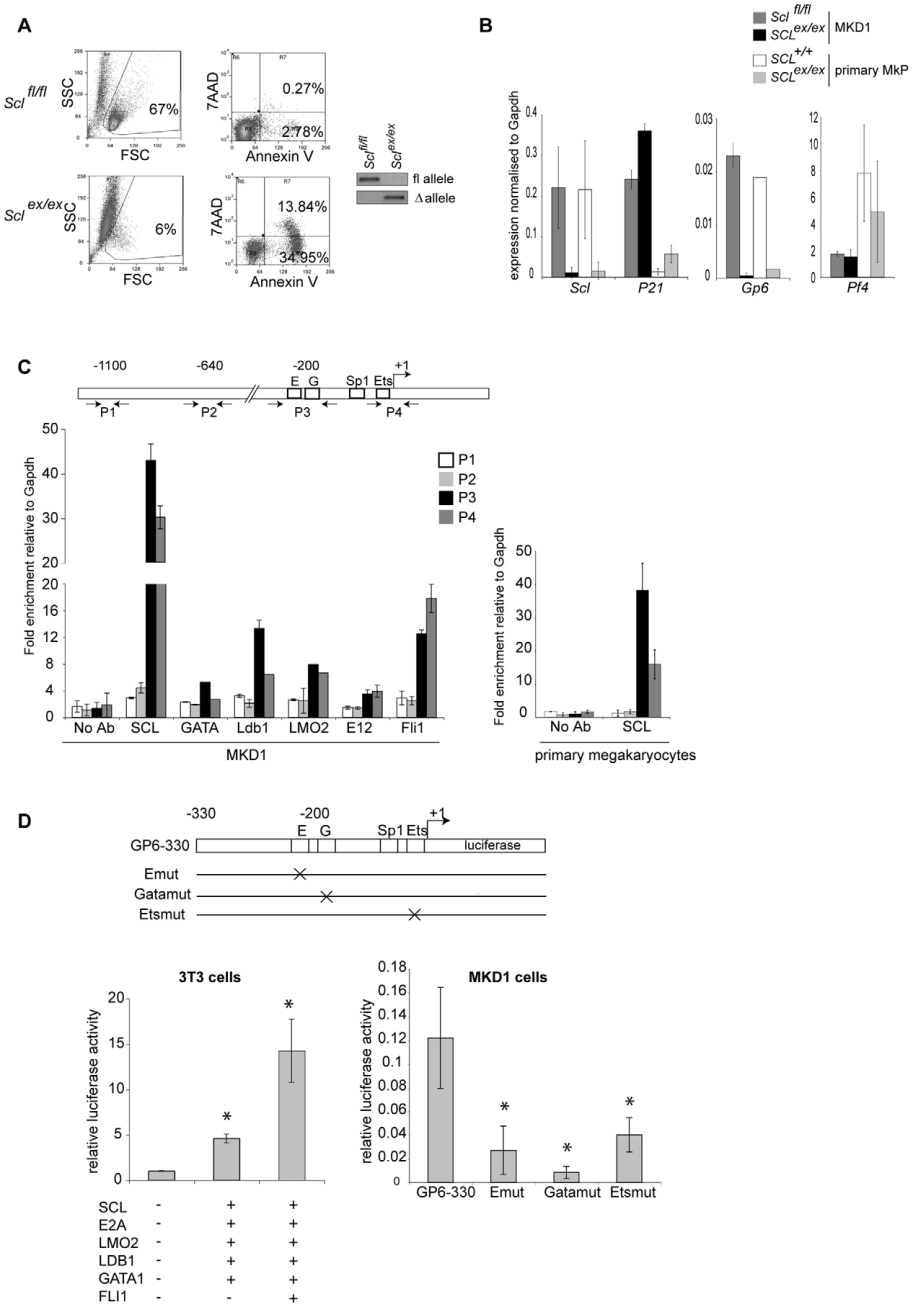
Functional and transcriptional analyses in MKD1 cells. (**A**) *Scl* excision induces apoptosis in MKD1 cells. Annexin V and 7AAD staining of MKD1 cells (*Scl^fl/fl^*) and upon Cre-mediated excision of the *Scl* floxed alleles (*Scl^ex/ex^*). (Left) Left panels, FSC and SSC parameters analysis showing the gate of viable cells. Rights panels, Annexin V/7AAD analysis. The percentages of necrotic (AnnexinV^+^ 7AAD^+^) and apoptotic cells (Annexin V^+^ 7AAD^−^) are shown. The data show one representative experiment out of three. (Right) PCR showing amplification of the floxed (fl) and excised (Δ) alleles in MKD1 cells (*Scl^fl/fl^*) and after Cre-mediated excision (*Scl^ex/ex^*). (**B**) Gene expression analysis by qRT-PCR of MKD1 and MkPs in SCL expressing cells (dark grey bars: MKD1 *Scl^fl/fl^*, white bars: *Cre;Scl^+/+^* MkPs) and upon Cre-mediated *Scl* excision; *Scl^ex/ex^* (black bars: MKD1 *Scl^ex/ex^*; light grey bars: *Cre;Scl^ex/ex^* MkPs). The *y*-axis represents the enrichment in cDNA sequences normalised to *Gapdh* gene control sequences. For MKD1, the histograms show the mean ± SD of 3 independent experiments, p<0.05. For the MkPs, the data show one representative experiment out of 2. (**C**) (Top) Schematic representation of the mouse *Gp6* proximal promoter. The location of the E box (E), Gata (G), Sp1 and Ets motifs is indicated, in bp relative to the transcription start site (+1). P1 to P4 show the location of the primer pairs designed for real-time PCR. Not to scale. (Bottom) ChIP analysis over the *Gp6* locus using material isolated from MKD1 cells (left) and primary megakaryocytes derived from 5-FU treated mouse (right). The antibodies used are indicated. The data show the mean ± SD of 3 independent experiments; enrichment over no antibody, *p<0.05. (**D**) Trans-activation assays in 3T3 (left) and MKD1 cells (right). Top, the luciferase gene is under control of a 330 bp fragment of the *Gp6* promoter. The reporter constructs with point mutations in the E box (Emut), Gata (Gatamut) and Ets motifs (Etsmut) are shown below. (Left) The graph shows relative luciferase activity measured in 3T3 cells transiently transfected with the luciferase reporter construct (GP6-330) and vectors expressing the indicated transcription factors. The mean ± SD of four independent experiments performed in duplicate is shown. *, p<0.05. (Right) The graph shows relative luciferase activity measured in MKD1 cells transiently nucleotransfected with the different luciferase reporter constructs as indicated. The mean ± SD of three independent experiments performed in duplicate is shown. *, p<0.05.

To carry functional investigations, we took advantage of the S*cl* floxed locus in MKD1 cells and of our recently described MK-specific *Scl^fl/fl^* mouse model [7] and compared the effects of *Scl* excision in MKD1 cells and primary MkPs. Confirming the critical role of SCL in MK differentiation [21], Cre-mediated excision of *Scl* floxed alleles triggered apoptosis in MKD1 cells (Figure 2A). Similarly to what we observed from *Scl*-deleted primary MkPs [7], *p21* (cell cycle inhibitor) expression was 1.5-fold increased in *Scl*-deleted MKD1 cells (Figure 2B). Likewise, a dramatic decrease in *Gp6* expression was observed from *Scl*-deleted MKD1 cells, *Scl*-deleted MkPs (Figure 2B) and *Scl*-deleted MKs [7], suggesting that SCL normally activates *Gp6* expression in immature (MkPs and MKD1) as well as mature megakaryocytes. As a control, *Pf-4* expression is unaltered in *Scl^ex/ex^* MKD1, MkPs (Figure 2B) and MKs [7]. In conclusion, our data demonstrate that *Scl* excision affects gene expression similarly in primary MkPs and MKD1 cells.

Finally, we analysed SCL-mediated transcriptional regulation of *Gp6* in MKD1 cells. SCL is part of a multiprotein complex (including E-proteins such as E12, LMO2, LDB1, and GATA1) that regulates megakaryocytic gene expression [8], [22]. Using chromatin immunoprecipitation (ChIP) assays, all members of this pentameric complex, as well as FLI1, an important regulator of megakaryopoiesis [23], were detected on the *Gp6* proximal promoter in MKD1 cells, on sequences containing E-box, GATA, Sp1 and Ets motifs (Figure 2C, left panel, regions P3 and P4). Importantly, the same sequences were bound by SCL in primary MKs (Figure 2C, right panel). In luciferase assays, co-expression of the SCL complex and FLI1 in 3T3 cells correlated with *Gp6* promoter transcriptional activity, while introduction of point mutations in the E-box, Gata and Ets motifs reduced *Gp6* promoter activity in MKD1 cells by 70, 90 and 60% respectively (Figure 2D), hence establishing the functional activity of this complex. Therefore, gene expression, ChIP and transcriptional analyses can be successfully carried out in MKD1 cells, opening the way to biochemical and functional studies.

To date, only three animal megakaryocytic cell lines have been reported [24]–[26]. Here, we describe the mES cell-derived MKD1 clone as a close model of mouse primary MkPs. These cells express very low levels of Mpl; restoring MPL expression in MKD1 cells may allow the cells to respond more specifically to TPO signalling and rescue their differentiation potential (as observed for the human cell lines UT7 [27] and 32D [28]) and providing an even more potent model of megakaryopoiesis.
